# Mixed model approach for IBD-based QTL mapping in a complex oil palm pedigree

**DOI:** 10.1186/s12864-015-1985-3

**Published:** 2015-10-15

**Authors:** Sébastien Tisné, Marie Denis, David Cros, Virginie Pomiès, Virginie Riou, Indra Syahputra, Alphonse Omoré, Tristan Durand-Gasselin, Jean-Marc Bouvet, Benoît Cochard

**Affiliations:** CIRAD, UMR AGAP (Genetic Improvement and Adaptation of Mediterranean and Tropical Plant Research Unit), Campus international de Baillarguet, 34398 Montpellier, France; Agriculture Department, SOCFINDO (PT Socfin-Indonesia), Medan, Indonesia; INRAB, CRAPP, Pobè, Bénin; PalmElit SAS, 34980 Montferrier sur Lez, France

**Keywords:** Pedigree, Identity by descent, Quantitative trait locus, Linear mixed model, Oil palm

## Abstract

**Background:**

*Elaeis guineensis* is the world’s leading source of vegetable oil, and the demand is still increasing. Oil palm breeding would benefit from marker-assisted selection but genetic studies are scarce and inconclusive. This study aims to identify genetic bases of oil palm production using a pedigree-based approach that is innovative in plant genetics.

**Results:**

A quantitative trait locus (QTL) mapping approach involving two-step variance component analysis was employed using phenotypic data on 30852 palms from crosses between more than 300 genotyped parents of two heterotic groups. Genome scans were performed at parental level by modeling QTL effects as random terms in linear mixed models with identity-by-descent (IBD) kinship matrices. Eighteen QTL regions controlling production traits were identified among a large genetically diversified sample from breeding program. QTL patterns depended on the genetic origin, with only one region shared between heterotic groups. Contrasting effects of QTLs on bunch number and weights reflected the close negative correlation between the two traits.

**Conclusions:**

The pedigree-based approach using data from ongoing breeding programs is a powerful, relevant and economic approach to map QTLs. Genetic determinisms contributing to heterotic effects have been identified and provide valuable information for orienting oil palm breeding strategies.

**Electronic supplementary material:**

The online version of this article (doi:10.1186/s12864-015-1985-3) contains supplementary material, which is available to authorized users.

## Background

Oil palm (*Elaeis guineensis* Jacq.) is a perennial allogamous species originating from Africa. It is the world’s leading crop for vegetable oil production (34.7 %, USDA statistics). A dynamic breeding sector has contributed to enhancing oil yields by 60 % in the last 50 years [1]. However, due to the increasing demand for edible oil and the diverse range of uses of palm oil, it is necessary to further increase oil yields to boost production in restricted cultivated areas. One main oil palm breeding scheme that is used was developed in the 1950s and is derived from a reciprocal recurrent selection (RRS) scheme, as described by Gascon & De Berchoux [2]. This breeding scheme was designed to take advantage of the heterotic effects on bunch production observed between Asian and African (heterotic group A and B, respectively) genetic backgrounds. The heterosis effect is the result of a better combination of yield underlying components, bunch number and weight, with group A palms producing a low number of big bunches and conversely for B palms, the two traits being negatively correlated [3]. Parents from both heterotic groups were assessed in large-scale field trials where candidate palms from full-sib families were progeny tested in A × B crosses. Through a better understanding of this heterosis effect, the selection of parents from heterotic groups could be enhanced so as to optimize the complementation of bunch production components.

QTL mapping could play a major role in making the selection process more efficient through the identification of the genetic basis of quantitative traits and the inclusion of this knowledge in breeding programs [4]. In oil palm, marker-assisted selection was proposed quite early to optimize the long breeding cycles, i.e. up to 20 years, but so far relatively few genetic studies have been published. QTL mapping has been used to investigate yield components [5–7], fatty acid composition [8, 9], sex ratio [10] or embryogenesis [11]. These studies involved different types of mapping population derived from one to four parents in intra- or inter-specific crosses, within *Elaeis guineensis* species or with its related species *Elaeis oleifera* respectively. However, because of large areas needed for palm tree evaluation, a limited number of individuals per cross are planted. Hence, most of these studies are limited by the insufficient population sizes, i.e. in the best cases around a hundred individuals but often much less, which can reduce the effectiveness of analysis and lead to inflated estimations of QTL effects [12]. QTL mapping contributions to breeding programs with other species are thus often fewer than expected [4, 13]. In addition to underpowered experimental design, genetic material in QTL mapping studies is often not very representative of the genetic diversity used in breeding programs, especially in populations derived from biparental crosses, which jeopardizes the effective transferability of the results. The recent development of new types of genetic material, such as *Arabidopsis* MAGIC [14] or AMPRIL [15] populations, or the maize NAM populatio[45nun [16], increases the number of alleles in segregation, while also striving to overcome this limitation. In oil palm, a study used this kind of multi-parental design by analyzing four connected full sib families between four parents [6], with simultaneous advantages of analyzing larger numbers of individuals and segregating alleles.

In agronomic species, an interesting way to improve the transferability of genetic knowledge and avoid additional costs of developing and phenotyping specific genetic material is to conduct *in silico* QTL mapping, i.e. based on available phenotypic and genetic data from commercial breeding programs [17]. This emerging opportunity gives rise to new issues in terms of experimental design and statistical approaches [18] and examples in different settings are still needed to assess its potential. In oil palm, progeny-based phenotypic evaluations of parents, with mostly known pedigree, have provided up to 10 years of data which is of interest for testing an *in silico* QTL mapping approach. Statistical methods for QTL mapping in pedigrees were first developed in human and animal genetics under high experimental design constraints. George *et al.* [19] proposed a two-step variance component approach based on mixed linear models that include IBD information at tested genetic positions. In plants, few studies report the implementation of this approach to map QTL, e.g. in wheat [20]. Statistical approaches based on linear mixed modeling generally allow flexibility for QTL testing, as shown in Van Eeuwijk *et al.* [21] where QTLs were mapped for hybrid performance in maize by testing QTL effects in both heterotic groups. Bayesian frameworks have also been developed to map QTLs in multiple related crosses [22, 23] and used successfully in apple [24] and cherry [25].

The aim of this study was to investigate the genetic architecture of production traits in two heterotic groups of oil palm. A QTL mapping approach was used to analyze bunch yield variation in the A × B population and to assess the complementation of yield underlying components, bunch weight and number, and their relationship with fruit bunch yield. To overcome conventional QTL mapping limitations that have been identified in oil palm and other perennial species, a promising pedigree-based approach was used to analyze traits involved in heterosis directly from current breeding program data.

## Methods

### Plant material

All palms analyzed belonged to families of the commercial oil palm (*Elaeis guineensis* Jacq.) breeding program of PalmElit, a CIRAD subsidiary and leading oil palm breeding company (www.palmelit.com). This breeding program is shared and conducted with partners, i.e. PT Socfin Indonesia (Indonesia) and INRAB (Benin). The 30852 palms with phenotypic evaluations belong to 478 crosses between 146 and 156 parents from heterotic groups A (GA) and B (GB), respectively. Most of the parents were planted in Pobè (INRAB, Benin), where the crosses were carried out. AxB crosses were planted in Aek Loba estate (PT Socfin Indonesia). Heterotic groups were based on the complementarity of yield components, bunch number and average weight, with GA palms having a low number of big bunches and GB palms having a high number of small bunches [26]. GA consisted of 132 palms from the “Deli” population derived from four oil palms planted in 1848 in Indonesia [27], while the remaining 11 palms originated from Angola. GB consisted of palms from La Mé (106, Côte d’Ivoire), Yangambi (23, Democratic Republic of the Congo), La Mé x Yangambi (5), La Mé x Sibiti (6, Democratic Republic of the Congo) and Nigeria (5).

Pedigree information was available for individuals in both heterotic groups (Additional file 1: Figure S1). However, for Deli individuals, the pedigree was known for a maximum of four generations. In this study, the Deli pedigree was reconstructed with MOLCOANC 3.0 software, as described in Cros *et al.* [28], to obtain kinship coefficients between all Deli palms.

### Phenotypic data

Phenotypic evaluations were conducted in 26 trials on 30782 A × B oil palms that had been planted on 350 ha at Aek Loba (Indonesia, SOCFINDO estate) between 1995 and 2000 in a study lasting 11 years. The experimental designs of the trials involved randomized complete block designs (RCBD) with five or six blocks and balanced lattices of ranks four or five. The data used in this study was from oil palms which had reached a mature stage 6 to 10 years after planting. Mature bunch harvests were performed every 10 days, and the bunch numbers and weights were recorded. Based on these data, three production traits were analyzed, i.e. fresh fruit bunch yield (FFB, kg/year), bunch number (BN, bunch/year) and average bunch weight (ABW, kg), which is the ratio between FFB and BN estimated annually.

A linear mixed model was designed to account for non-genetic (trial, block) and genetic (general combining ability (GCA) and specific combining ability (SCA)) effects and adjusted to the data:

1 $$ Y=X\beta +{Z}_1u+{Z}_2s+{Z}_A{g}_A+{Z}_B{g}_B+e $$

where *Y* is a *n* × 1 observation vector of production traits (*n* = 30852) averaged over 4 years in the A × B population, *X* is a *n* × *m* design matrix relating observations to trial fixed effects *β* with *β* being a *m* × 1 vector (*m* = 26), *Z*_1_ is a *n* × *b* design matrix relating observations to block random effects *u* ~ *N*(0, *Iσ*_*u*_^2^) with *u* being a *b* × 1 vector (*b* = 677), *Z*_2_ is a *n* × *c* design matrix relating observations to SCA random effects *s* ~ *N*(0, *Iσ*_*s*_^2^) with *s* being a *c* × 1 vector (*c* = 478), *Z*_*A*_ and *Z*_*B*_ are *n* × *q*_*A*_ and *n* × *q*_*B*_ design matrices relating observations to GCA random effects for heterotic groups A and B, *g*_*A*_ ~ *N*(0, *A*_*A*_*σ*^2^*a*_*A*_ ) and *g*_*B*_ ~ *N*(0, *A*_*B*_*σ*^2^*a*_*B*_ ) respectively, with *g*_*A*_ and *g*_*B*_ being *q*_*A*_ × 1 and *q*_*B*_ × 1 vectors, respectively (*q*_*A*_ = 146 and *q*_*B*_ = 156), and *e*is the *n* × 1 vector of residual effects ~ *N*(0, *Iσ*_*e*_^2^). *I* is an identity matrix and *A*_*A*_ and *A*_*B*_ are the pedigree-based kinship matrices of heterotic groups A and B, respectively.

Calculation of heritabilities and genetic correlations were based on variances estimated by the linear mixed model (1). Narrow-sense heritabilities (h^2^) of each trait were obtained as the ratio of the variance of general combining abilities, for both groups A and B, to the total phenotypic variance of crosses according to the formula:

$$ {h}_{A/B}^2=\frac{\sigma^2{a}_{A/B}}{\sigma_b^2+{\sigma}_s^2+{\sigma}^2{a}_A+{\sigma}^2{a}_B+{\sigma}_e^2} $$

Genetic correlations (*r*_*g*_) between traits T_1_ and T_2_ in each heterotic group were calculated using covariances and variances estimated in a multivariate linear mixed model, based on model (1). The model had the form:

2 $$ \left[{}_{T_2}^{T_1}\right]=\left[{{}_0^X}_X^0\right]\kern0.5em \left[{}_{\beta_{T2}}^{\beta_{T1}}\right]+\left[{{}_0^{Z_1}}_{Z_1}^0\right]\kern0.5em \left[{}_{u_{T2}}^{u_{T1}}\right]+\left[{{}_0^{Z_2}}_{Z_2}^0\right]\left[{}_{S_{T2}}^{S_{T1}}\right]+\left[{{}_0^{Z_A}}_{Z_A}^0\right]\left[{}_{{g_A}_{T_2}}^{g_{A{T}_1}}\right]+\left[{{}_0^{Z_B}}_{Z_B}^0\right]\left[{}_{g_{B_{T2}}}^{g_{{B_T}_1}}\right]+\left[{}_{e_{T_2}}^{e_{T_1}}\right] $$

GCA were structured as:

$$ \left[\begin{array}{c}\hfill {g_A}_{T_1}\hfill \\ {}\hfill {g_A}_{T_2}\hfill \end{array}\right]\sim N\left(0,\left[\begin{array}{cc}\hfill {\sigma}^2{a}_{A_{T_1}}\hfill & \hfill Co{v}_A\left({T}_1,{T}_2\right)\hfill \\ {}\hfill Co{v}_A\left({T}_1,{T}_2\right)\hfill & \hfill {\sigma}^2{a}_{A_{T_2}}\hfill \end{array}\right]\otimes {A}_A\right) $$

$$ \left[\begin{array}{c}\hfill {g_B}_{T_1}\hfill \\ {}\hfill {g_B}_{T_2}\hfill \end{array}\right]\sim N\left(0,\left[\begin{array}{cc}\hfill {\sigma}^2{a}_{B_{T_1}}\hfill & \hfill Co{v}_B\left({T}_1,{T}_2\right)\hfill \\ {}\hfill Co{v}_B\left({T}_1,{T}_2\right)\hfill & \hfill {\sigma}^2{a}_{B_{T_2}}\hfill \end{array}\right]\otimes {A}_B\right) $$

where *Cov*_*A*_(*T*_1_, *T*_2_) and *Cov*_*B*_(*T*_1_, *T*_2_) are additive genetic covariances.

Residual effects were structured as:

$$ \left[\begin{array}{c}\hfill {e}_{T_1}\hfill \\ {}\hfill {e}_{T_2}\hfill \end{array}\right]\sim N\left(0,\left[\begin{array}{cc}\hfill {\sigma}^2{{}_e}_{T_1}\hfill & \hfill Co{v}_e\left({T}_1,{T}_2\right)\hfill \\ {}\hfill Co{v}_e\left({T}_1,{T}_2\right)\hfill & \hfill {\sigma}^2{{}_e}_{T_2}\hfill \end{array}\right]\otimes I\right) $$

where *Cov*_*e*_(*T*_1_, *T*_2_) is residual covariance.

Genetic correlations (*r*_*g*_) were calculated according to the formula:

$$ {r}_{gA/gB}=\frac{Co{v}_{A/B}\left({T}_1,{T}_2\right)}{\sigma_{A/B\ }{T}_1\times {\sigma}_{A/B\ }{T}_2} $$

Phenotypic correlations (*r*_*p*_) were estimated in the A × B population via the Pearson correlation coefficient.

### Molecular data and genetic map construction

The genotyped population consisted of palms from both heterotic groups which were parents of the crosses evaluated in the genetic trials. Palms were genotyped with 388 SSR markers developed in different studies [29–31].

Total genomic DNA was extracted from freeze-dried leaf samples of each progeny and parent using the commercial kit NucleoSpin 96 plants II (Macherey-Nagel, Germany) in accordance with the manufacturer’s protocol. Genomic DNA concentrations were estimated with a spectrometer (Infinite® 200 PRO NanoQuant®, Switzerland). Microsatellite fragment amplification was performed in a 384-well plate with 25 ng of DNA in a 10 μl final volume containing 1 U of Taq DNA polymerase, 1 μl of 10x buffer (10 mM Tris (pH 8.3), 50 mM KCl, 1.5 mM MgCl2, 0.001 % (w/v) glycerol), 2 mM dNTP, 0.6 mM MgCl_2_, 0.08 μM of M13-tailed primer, 1 μM of the reverse primer, 1 μM of M13 primer-fluorescent dye VIC, PET, NED or FAM (Applied Biosystems, USA). The PCR conditions were as follows: initial denaturation at 94 °C for 5 min, followed by 35 cycles alternating 30 s of denaturation, 1 min 15 s of hybridation at annealing temperature, 1 min 30 s of extension, and ending with 30 min of final elongation at 72 °C. PCR products were pooled. We used 2 μl of the pooled PCR product with 10 μl of size standard GeneScan ™-600 LIZ ® at 0.0012 % in Hi-Di™ formamide for migration on a 3500xL Genetic Analyzer (Applied Biosystems, USA).

GeneMapper© V4.1 (Applied Biosystems, USA) software was used to determine allele sizes.

A consensus linkage map (Additional file 1: Figure S2) based on the pedigree of both groups A and B was constructed with CRIMAP version 2.4 [32], as described in [33]. The genetic map was drawn using MapChart software [34].

### QTL mapping

QTL mapping was performed following the two-step variance component approach described in George *et al.* [19]. Van Eeuwijk *et al.* [21] presented an adapted approach to identify QTLs in a maize hybrid program that tested parents from heterotic groups like in oil palm. The approach presented here was extended to an outbreeding population for which genotyping data were available only on the parents of both heterotic groups A and B and phenotypic data only on A × B individuals. The first step involved computation of IBD kinship matrices over a grid of genetic positions. The second step consisted of QTL presence tests over the grid of genetic positions by comparison of various linear mixed models.

#### IBD matrix computation

QTL mapping using a variance component approach requires identity-by-descent (IBD) relationship matrices as variance-covariance matrices for testing QTL effects at each genetic position. An IBD analysis using SimWalk2 software [35] was conducted to estimate the IBD matrices formed by empirical kinship coefficients for each pair of palm trees present in group A and B pedigrees. Simwalk2 implements a Bayesian framework using a Markov chain Monte Carlo (MCMC) algorithm to select the most likely genetic descent graph depicting inheritance patterns within pedigrees [36]. Empirical kinship coefficients were calculated at every marker position and over a 3 cM interval grid, leading to 922 evaluation points for QTL presence testing using IBD analysis in Simwalk2.

#### Statistical modeling

The model (1) (Phenotypic data section) was used as a null model for QTL mapping. Two other models were designed to test for the QTL presence in each heterotic group by adding a random QTL effect to model (1). A genome scan was performed by fitting models for groups A and B at k genetic positions used to compute IBD kinship matrices testing for the QTL presence. The models had the form:

3 $$ Y=X\beta +{Z}_1u+{Z}_2s+{Z}_A{g}_A+{Z}_B{g}_B+{Z}_A{v}_{A,k}+e $$

4 $$ Y=X\beta +{Z}_1u+{Z}_2s+{Z}_A{g}_A+{Z}_B{g}_B+{Z}_B{v}_{B,k}+e $$

where *v*_*A*,*k*_ and *v*_*B*,*k*_ are *q*_*A*_ × 1 and *q*_*B*_ × 1 vectors of QTL random effects at the k^th^ genetic position in heterotic group A and B respectively (*q*_*A*_ = 146 and *q*_*B*_ = 156), with *v*_*A*,*k*_ ~ *N*(0, *A*_*A*,*k*_*σ*^2^*a*_*A*,*k*_) and *v*_*B*,*k*_ ~ *N*(0, *A*_*B*,*k*_*σ*^2^*a*_*B*,*k*_). *A*_*A*,*k*_ and *A*_*B*,*k*_ are the IBD kinship matrices at the k^th^ genetic position, calculated in groups A and B, respectively.

The log-likelihood ratio test (LRT) was performed to compare models (3)/(4) and (1) at each k position. As the distribution of LRT is unknown under the null hypothesis [19], permutations were performed to estimate significance thresholds (see next paragraph). A QTL was declared significant if the LRT exceeded the threshold, and sets of putative QTLs in both heterotic groups were constituted.

According to a multiple QTL mapping strategy [37] to better account for the QTL variance of QTL elsewhere in the genome when testing a genetic position, a second genome scan was then performed with updated null (5) and test (6)/(7) models that incorporated putative QTL random effects for both heterotic groups:

5 $$ Y=X\beta +{Z}_1u+{Z}_2s+{Z}_A{g}_A+{Z}_B{g}_B + {\displaystyle \sum_{c\  in\ {C}_A\ }}{Z}_A{v}_{A,c}+{\displaystyle \sum_{c\  in\ {C}_B\ }}{Z}_B{v}_{B,c} + e $$

6 $$ Y=X\beta +{Z}_1u+{Z}_2s+{Z}_A{g}_A+{Z}_B{g}_B+{\displaystyle \sum_{c\  in\ {C}_A\ }}{Z}_A{v}_{A,c}+{\displaystyle \sum_{c\  in\ {C}_B\ }}{Z}_B{v}_{B,c}+{Z}_A{v}_{A,k}+e $$

7 $$ Y=X\beta +{Z}_1u+{Z}_2s+{Z}_A{g}_A+{Z}_B{g}_B+{\displaystyle \sum_{c\  in\ {C}_A\ }}{Z}_A{v}_{A,c}+{\displaystyle \sum_{c\  in\ {C}_B\ }}{Z}_B{v}_{B,c}+{Z}_B{v}_{B,k}+e $$

Where *C*_*A*_ and *C*_*B*_ are subsets of genetic positions where putative QTLs were identified in the first genome scan for groups A and B, respectively. To test the QTL presence at genetic positions within a 20 cM window centered on *C*_*A*_ and *C*_*B*_ positions, the random putative QTL effect at the considered positions was removed in the null and test models (5) and (6)/(7), respectively.

As for the first genome scan model, (6)/(7) and (5) were compared using LRT and QTLs were declared significant if the LRT exceeded the threshold determined by permutation. Approximate confidence intervals of QTL location were estimated by 1-LRT support interval calculation, i.e. the interval in which LRT is within 1 units of its maximum [38]. Final multi-QTL models were fitted for each production trait incorporating random effects for all QTLs identified in the second genome scan. Best linear unbiased predictors (BLUP) were obtained as predictors of additive value of each individual at a given QTL. BLUPs were plotted on the pedigree graph grouping all tested parents using Pedimap software [39].

All linear mixed effect models were estimated by REML using the R-ASReml package (Butler et al., 2009) for R [40].

#### LRT threshold calculation

LRT thresholds used to determine the presence or absence of QTLs at the tested genetic positions were set independently for each heterotic group, each production trait and for the first and second genome scans. The threshold calculation was based on the distribution of the LRT under the hypothesis of the absence of QTLs in the genome. Null distributions were obtained by permutations [41], conducting genome scans with de-correlated QTL effects from phenotypes without affecting other effects correlations. To do that, vectors of IBD coefficients constituting the IBD kinship matrices associated to QTL effects, *A*_*A*,*k*_ and *A*_*B*,*k*_ in the scan model (see statistical modeling section), were randomly shuffled before each replicate of genome scan, whereas all other components were unchanged comparing to the null model. 1000 and 500 replicates were obtained to determine each threshold for the first and second genome scans of QTL mapping process respectively, for each trait and heterotic group (Additional file 1: Figure S3). For each replicate, the maximum LRT value obtained was recorded to construct the distribution of maximum LRT value under the null hypothesis. The 95 % percentile was calculated based on this distribution to determine a 5 % LRT threshold. To save computing time, a reduced density of evaluation points was tested for each replicate genome scan, with 1 out of 25 and 50 positions for the first and second genome scan of QTL mapping process respectively, which led to 40,000 and 10,500 tests of null hypothesis, respectively, to obtain the null distribution. The testing frame was shifted for each permutation sample to ensure that all genetic positions were assessed.

#### Assessment of QTL pleiotropic effects

18 QTL regions were identified based on LRT thresholds and the approximate confidence intervals of QTL location. Linear mixed models of the form (5) including effects for the 18 QTL regions were adjusted for each production variable. Additive QTL effects were predicted for parents of corresponding heterotic groups and Pearson correlation coefficients between them were calculated for each pair of production variables.

## Results

### Variation for production traits in AxB population

**Fig. 1:**
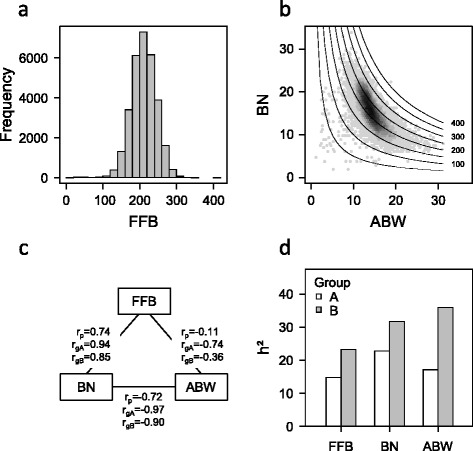
Production trait variations in oil palm A × B population. **a** Distribution of fresh fruit bunch weight (FFB). **b** Relationship between average bunch weight (ABW) and bunch number (BN) in an A × B population. The grey scale indicates the density of points with similar BN and ABW values. Isoproduction curves are drawn with corresponding FFB values given on the right of the curves. **c**Phenotypic correlations (r_p_) and genotypic correlations in heterotic groups A (r_gA_) and B (r_gB_) between FFB, BN and ABW. **d** Narrow sense heritability (h^2^) for the three production traits, i.e. FFB, BN and ABW, estimated from A × B individuals

Fresh fruit bunch yield (FFB) and underlying components bunch number (BN) and average bunch weight (ABW) were normally distributed within the hybrid population (30852 palms) derived from crosses between palms of heterotic groups A and B, and ranged from 6 to 411 kg/palm/year, 2 to 34 bunches/year and 1.2 to 31.4 kg, respectively (Fig. 1). Coefficients of variation were around 16 % for FFB and ABW and 22 % for BN. In the A × B population, BN and ABW were highly and negatively correlated and FFB was highly and positively correlated with BN, while it was negatively correlated with ABW (Fig. 1c). Genetic correlations showed the same pattern, with closer correlations found in the heterotic group A than B (Fig. 1c). Consequently, the isoproduction curves on Fig 1b show that the higher FFB values were mainly reached with high BN rather than high ABW.

Heritability based on estimation of variance components with only pedigree information ranged from 0.15 for FFB in heterotic group A to 0.36 for ABW in heterotic group B (Fig. 1d). Heritability was lower in heterotic group A than in heterotic group B for the three production traits, with a lower additive genetic variance found in group A (Fig. 1d).

### QTL mapping for production traits

**Fig. 2:**
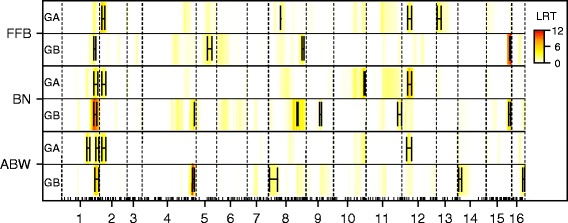
Genetic determinisms of production trait variation in an oil palm A × B population. Heat map of a log-likelihood ratio test (LRT) testing the presence of a QTL in group A (GA) and B (GB) for the three production traits (FFB, BN and ABW). LRT are plotted along the 16 oil palm linkage group, and dashes on X-axis represent markers present on the genetic map. Bars on significant QTL positions represent approximate confidence intervals of QTL location

Figure 2 shows a heat map of the log-likelihood ratio test (LRT) for the second genome scan of QTL mapping process (see Material and Methods) in each heterotic group for the three production traits. Eighteen significant QTL regions were identified, i.e. seven in group A and eleven in group B. The QTL regions were distributed in 14 linkage groups, with linkage group 1 and 8 harboring two and four QTL regions, respectively (Fig. 2). QTL confidence intervals spanned 14.5 cM on average, ranging from 2 to 42 cM (Fig. 2).

The final QTL models consisted of eight QTLs for FFB, ten for BN and nine for ABW, jointly accounting for 50, 70 and 75 % of the phenotypic variance of the hybrid population, respectively (Table 1). The residual variance was 18 to 29 % lower with the final QTL models than with the pedigree-based model (1) (Table 1). Pedigree variance components in the final QTL models were all below 1 % of the phenotypic variance, except for the group B pedigree variance component for FFB (Table 1).

**Table 1:** Variances of genetic model components for three production traits in oil palm

		Variance	
Traits	Components	PED	PED + QTL
FFB			
	A_GCA	14.72	0.15
	A_2@12	-	5.29
	A_8@59	-	4.08
	A_12@33	-	6.71
	A_13@7	-	3.69
	B_GCA	23.19	1.89
	B_1@152	-	2.12
	B_5@69	-	3.68
	B_8@168	-	16.51
	B_15@117	-	8.89
	SCA	2.65	1.74
	Non genetic	3.86	2.94
	Residuals	55.58	42.31
BN			
	A_GCA	22.67	0.59
	A_1@163	-	5.55
	A_2@18	-	2.03
	A_10@151	-	2.08
	A_12@30	-	12.55
	B_GCA	31.60	0.00
	B_1@159	-	4.18
	B_4@253	-	23.79
	B_8@137	-	4.40
	B_9@69	-	4.88
	B_11@172	-	6.23
	B_15@117	-	4.27
	SCA	2.72	1.60
	Non genetic	2.40	1.56
	Residuals	40.60	26.29
ABW			
	A_GCA	17.04	0.11
	A_1@123	-	1.90
	A_1@167	-	1.99
	A_2@19	-	1.34
	A_12@30	-	10.73
	B_GCA	35.85	0.00
	B_1@167	-	6.80
	B_4@252	-	44.60
	B_8@24	-	1.07
	B_14@9	-	0.90
	B_16@57	-	5.88
	SCA	3.33	1.46
	Non genetic	2.63	1.39
	Residuals	41.15	21.84

Variances are presented in percentage of total variance for the model without QTL effects (PED) and for the final selected QTL model (PED + QTL). QTL names are formed by heterotic group ID (A or B), the number of linkage group and the position in cM

*FFB* Fresh fruit bunch yield

*BN* Bunch number

*ABW* Average bunch weight

*GCA* General combining ability

*SCA* Specific combining ability

A major QTL was found for group B on the bottom of linkage group 4, accounting for 21 and 44 % of the phenotypic variance of BN and ABW, respectively (Fig. 2). However, this region was not significant for FFB due to the opposite effects on BN and ABW. The strongest QTL found in group A was located on the top of linkage group 12 and it accounted for 7, 12 and 11 % of the phenotypic variance of FFB, BN and ABW, respectively (Fig. 2).

### Pleiotropic effects of QTLs

**Fig. 3:**
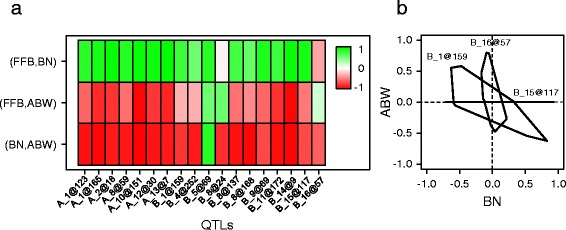
QTL effects on the three production traits in oil palm. **a** Correlation between production variables at QTL positions, measured as the Pearson correlation coefficient between BLUPs of the random QTL effects for each variable pair. The color scale indicates the strength of the correlation, with green and red being perfect positive and negative correlations, respectively. **b** Relationships between QTL effects for bunch number (BN) and average bunch weight (ABW) in heterotic group B parents are presented using the convex hull encompassing the scatter plot of parental BLUP values (n = 146). Convex hulls are drawn for BLUPs at three QTL positions on linkage group 1 (B_1@159), 15 (B_15@117) and 16 (B_16@57)

Correlations obtained between production traits at the hybrid population level indicated that common genetic regions should control several traits. To assess the prevalence of these pleiotropic QTLs, correlations between additive values of each parent at a given QTL were calculated for the 18 QTL regions identified for the three production traits (see Material and methods). Figure 3a shows the correlation patterns among pairs of traits, heterotic groups and QTLs. In heterotic group A, all correlations were high and positive between FFB and BN, and negative between ABW and the other two traits (Fig. 3a). The same pattern was observed in eight of the eleven QTLs identified in heterotic group B (Fig. 3a). Two of the three remaining QTLs, i.e. B_8@24 and B_16@57, had opposite effects on BN and ABW, but the effects on ABW were positively correlated with the effects on FFB (Fig. 3a). B_5@69 was the only QTL that had positive effects on the three production traits (Fig. 3a), but no significant effect was found on BN and ABW (Table 1).

Figure 3b illustrates the different types of QTL found in heterotic group B. QTL B_1@159 had opposite and significant effects on BN and ABW, like the majority of QTLs found (Fig. 3b), which could explain the correlations obtained in the hybrid population. However, QTLs that had trait-specific effects were found, on BN exclusively (B_15@117, Fig. 3b) or with superior effects on ABW, while the effect on BN was non-significant (B_16@57, Fig. 3b).

### Overlapping of QTLs between heterotic groups

**Fig. 4:**
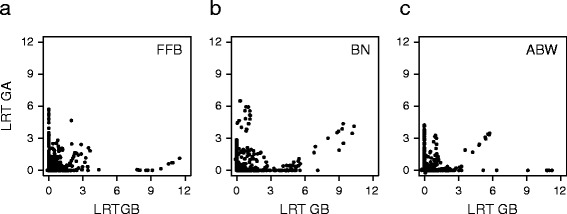
Co-localization of QTLs between oil palm heterotic groups. Log-likelihood ratio tests (LRT) for QTL mapping are plotted in heterotic group A (GA) versus heterotic group B (GB) for the three production traits, i.e. fresh fruit bunch (FFB, **a**), bunch number (BN, **b**) and average bunch weight (ABW, **c**)

To assess the complementarity of QTL patterns as a possible cause of the heterosis observed in the hybrid population, the findings of a log-likelihood ratio test (LRT) for the presence of additive QTLs in each heterotic group at all genomic evaluation points were plotted for group A versus group B (Fig. 4). For FFB, point locations mainly along the X and Y axes indicated that the QTL patterns differed between the two heterotic groups (Fig. 4a). On the contrary, for BN and ABW, points near the diagonal indicated overlapping of QTLs for these traits between the two heterotic groups (Fig. 4b and c). Among the 18 QTLs in the final model, these overlapping QTLs were found only on the bottom on linkage group 1 (1@165 for group A and 1@159 for group B, Fig. 2), but some others reached the significance threshold in only one of the two groups (linkage group 12, Fig. 2).

## Discussion

### Variance component approach for QTL mapping

QTL mapping is interesting when assessing perennial species [4], but biological and economic issues can hamper its use even for major crop species such as oil palm for which genetic studies are scarce. As proposed by Parisseaux and Bernardo [17], *in silico* mapping, i.e. based on data from current breeding programs, has many combined advantages. No extra costs are required to develop and phenotype specific mapping populations and this approach enables screening a high number of individuals and alleles, increasing relevance for the breeding process. Linear mixed model approaches used to map QTLs in such genetic material are flexible and powerful [42]. In the present study, this allowed us to test for the presence of QTLs in two heterotic groups, as proposed in maize by van Eeuwijk *et al.* [21], and to account for confounding nongenetic effects and polygenic effects, which can be important in such large trials with different genetic backgrounds. Moreover, one of the interesting benefits specific to this study was the possibility of linking phenotypes that were recorded exclusively in A × B individuals to genotypes available only for parents and grand-parents in some cases. Thus it enabled testing the presence of QTLs in both heterotic groups in a single model without first estimating breeding values of genotyped individuals. By genotyping only parents and grandparents, recombination events at the AxB individuals were not taken into account. This could limit the accuracy of the position of QTL and their confidence intervals which are mainly determined by the number of recombination events analyzed. However, the number of individuals genotyped, more than 300, is greater than the population sizes commonly found in oil palm and allowed, with moderate genotyping cost, to conduct QTL mapping based on a very high amount of phenotypic data, thus enhancing the detection power. In future studies, the flexibility of a mixed model framework should allow us to easily extend this approach to test environmental responses and dominance or epistasis effects.

In a two-step variance component approach, QTL mapping is influenced by the precision of IBD kinship matrices that highlight allele identities between individuals and recombination events between genetic positions. Marker and pedigree information were combined in multipoint analyses using Simwalk2 Bayesian software to estimate these matrices [35], which allowed us to handle such large and complex pedigrees. The pedigree information quality is a key factor in this step. Unlike the group B pedigree, which is well known for the different genetic origins that compose it, the group A pedigree is not fully known in higher generations. MOLCOANC software [28] was used to reconstruct the pedigree, and QTL mapping using the reconstructed pedigree identified two additional QTLs compared to the raw pedigree (Additional file 1: Figure S4). One of them on linkage group 12 is highly supported by a QTL found in the same location for the same traits in a multiparental population involving related palm trees as parents [6].

### Comparison to previous results in oil palm

Few other studies have aimed at mapping QTLs for the same production traits, despite frequent limitations for collecting data for a number of individuals that would be optimal for QTL mapping. Rance et al. [5] first attempted to map QTLs based on 84 individuals from an F2 population derived from a single palm selfing progeny. A saturated genetic map could not be obtained, and three putative QTLs were identified, one for each of three production traits, i.e. FFB, BN and ABW. More recently two studies investigated production traits in F1 populations based on 208 individuals [10] and 52 individuals [7]. The latest involved very few individuals and identified two QTLs for FFB and one for ABW, but none for BN. Ukoskit et al. [10], using a greater number of individuals, identified three QTLs for FFB. Three, two and five QTLs were found for FFB, BN and ABW, respectively, in a multiparental population pooling 299 individuals from an incomplete factorial design involving two parents of each heterotic group [6]. The two-step variance component approach used in this study identified eight, ten and nine QTLs for FFB, BN and ABW, respectively, and some other suggestive QTLs that were just below LRT threshold. The higher detection power achieved in our study compared to previous works was likely due to the higher genetic variation screened through pedigree based analysis. Analysis in F1 from single crosses, especially in oil palm for which our commercial elite genotypes are partially inbreed, screen a very restricted genetic basis and raises the risk that palms used as parents had homozygous positive alleles at many QTLs. Although the multi-parent design overcame this issue, it was less efficient than pedigree-based analysis. Moreover, the difference in detection power could be linked to the higher number of individuals in the mapping population, at least compared to studies that have fewer. The number of genotypes upon which the QTL mapping was based was actually not very high (around 150 in each heterotic group) as only the parents were genotyped, but the general combining abilities of these individuals were estimated very precisely via numerous progeny tests, with an accuracy of 0.9 [43].

CIRAD microsatellite marker resources have been widely used in oil palm genetics since the publication of a reference genetic map [29] that facilitated comparison of QTL mapping results between different studies. Among the 18 QTL regions identified in this study, eight were found by other authors for the same traits. Billotte *et al.* [6] identified the set of QTLs closest to our, with five shared regions as well as three with suggestive QTLs. This was not surprising as the parents derived from their factorial design were founders in the pedigrees of the group A and B individuals used in our study. The co-localization of pedigree-based QTLs with those found in previous studies in related or unrelated populations confirms the ability of the two-step variance component approach to identify regions that control production traits.

### Determinism of heterotic effects on fresh fruit bunch yield

The implementation of the large genetic trial used in this study follows the RRS with progeny testing of parents, motivated by the observations of increased FFB in A × B palms compared to the maximal value of intra-group crosses [3]. This heterosis effect might be explained by the better combination of underlying production traits, i.e. bunch number and average weight, that exhibited marked differences between heterotic groups, with group A palms having a low number of big bunches and group B palms having a high number of small bunches. Heterosis is an important phenomenon in plant breeding, especially in crops that exploit it through commercial hybrid production, but the details of its molecular determinism are still not well known. Dominance through complementation of inferior alleles and over dominance are the main mechanisms proposed without being mutually exclusive [44]. In maize, a QTL mapping approach on large pedigree of heterotic groups showed that complementation of positive alleles in the hybrids could be an important factor [21]. The low occurrence of collocations of QTLs between heterotic groups noted in the present study, only one among 18 significant QTL regions, confirms that both heterotic groups are genetically distant and suggests that complementation of favorable alleles would also be part of the phenomenon in oil palm. However, the experimental design used in this study was not adequate to identify QTL fixed for alternative alleles in heterotic groups and could have a major role on heterosis, because QTL effects were tested within heterotic groups and were identified only if the QTL was polymorphic within the group.

### Inclusion of QTL information in oil palm breeding programs

Our QTL mapping approach based on data from current breeding programs overcame usual ineffective analysis problems by pooling a sufficient amount of data, and the identified QTLs are highly relevant for integrating information for breeding. Moreover, the incomplete factorial design allowed us to obtain estimates of QTL allele effects from a wide range of genetic backgrounds, thus enhancing the transferability to breeding programs. However, as mentioned by Würschum [18], a validation step is necessary in marker-assisted selection to avoid the use of false-positive or overestimated QTLs, a problem that can arise due to biases in the statistical design. QTLs can be validated in independent oil palm pedigrees in other genetic trials to assess the stability of QTLs in different genetic or environmental settings, as has been done in other perennial species [45, 46]. An alternative approach would be to use the A × B population, described in this study, to validate QTLs at the progeny level by genotyping a high number of A × B individuals with markers identified at QTL locations. In addition, genotyping directly A × B individuals in several related progenies would offer two advantages: to give the statistical power for testing QTL effects conditionally of genetic background, and so detect dominance or epistasis effects between heterotic group A and B alleles, and to increase the number of recombination events in the pedigree analyzed in regions of interest, which combined with an increased genotyping density of these regions would reduce the confidence interval around QTLs. The latter would be interesting for QTLs identified in less well covered regions (e.g. top of linkage group 14) caused by difficulties in genetic map construction [33].

For the QTL validation or marker-assisted selection process, target QTL genotypes of pedigree members must be known in order to be able to design efficient crosses that test and combine segregating QTLs. The variance component approach modeled QTL effects as a random term, and estimates of the additive value of each individual at a given QTL were obtained with BLUPs. Despite the normal distribution of BLUPs for QTL effects, the segregation in full-sib families and alleles carried by pedigree members could be inferred qualitatively, e.g. by looking at the pedigree with projected BLUP values (Additional file 1: Figure S5). The Bayesian approach implemented in FlexQTL software offers an interesting way to predict the QTL genotype [23]. The presence of biallelic QTLs in the genome are tested simultaneously with QTL alleles carried by founders by inference on QTL genotype probabilities for any members of the pedigree based on pedigree, marker and phenotype data.

Such robust information about QTLs and their segregation in genetic backgrounds used in a breeding scheme could be integrated in the RRS to favor complementation of QTLs for production traits and thus to enhance the heterotic effects. As shown in Additional file 1: Figure S5, QTL segregation in heterotic groups differed depending on the genetic origins, e.g. La Mé and Yangambi, and recombination between these genetic origins could lead to improvements within heterotic groups by combining favorable alleles. Conventional pyramiding of interesting QTL approaches could be a way to obtain in few generations improved parents in each heterotic group. Although pyramiding could be efficient in the case of traits with simple genetic determinism, genomic selection is becoming an attractive approach for traits with genetic architecture following an infinitesimal model, especially in species with long breeding cycles and biological constraints. Various authors have proposed the inclusion of genetic architecture information to prioritize genomic regions in the estimation of breeding value [47–49]. The benefits of a genomic selection approach was evaluated using data from the same oil palm breeding program [43] and our results provide an opportunity to test, based on empirical data, a combined QTL and genomic selection approach to achieve efficient marker-assisted selection.

## Conclusions

18 QTL regions controlling production traits in oil palm were identified using data from current breeding programs analyzed with an efficient pedigree-based approach. Pleiotropic QTL regions with distinct patterns between and within oil palm heterotic groups were identified and provide valuable information for orienting oil palm breeding strategies.

## Additional file

Additional file 1: Figure S1. Pedigree of oil palm heterotic groups A (A) and B (B) individuals related to A×B progenies providing phenotypic data. Palm trees from group A originate from populations Deli (DELI) and Angola (AN), those of group B originate from populations La Mé (LAME), Yangambi (YBI), Nigeria (NI) and Sibiti (SI). Palm trees used as parents of A×B progenies are in white, and for group A pedigree, palm trees surrounded with red were reconstructed using MOLCOANC software. **Figure S2.** Oil palm consensus genetic map obtained from recombination information in group A and B pedigrees. Linkage groups are numbered according to Billotte *et al.* (2005, 2010). Map distance is given on the left in centimorgans (cM) and marker names on the right. **Figure S3.** Null distribution of log-likelihood ratio test in oil palm group A (green) and B (blue) for first (LRT_1,A) and second (LRT_2,B) genome scan for fresh fruit bunch weight (FFB), bunch number (BN) and average bunch weight (ABW) in heterotic group A (green lines) and B (blue lines). **Figure S4.** Log-likelihood ratio test (LRT) profile obtained in the first genome scan (see Material and Methods) using known (black lines) or reconstructed (green lines) pedigree of oil palm heterotic group A individuals. **Figure S5.** Example of QTL segregation in oil palm pedigree. Additive effects on bunch number for pedigree term (A,D) and QTLs on linkage group 9 at 69 cM (B,E) and on linkage group 15 at 117 cM (C,F) are projected on two subsets of heterotic group B pedigree, one centered on La Mé LM10T individual (A-C) and one grouping most of Yangambi individuals (D-F) . Red to green scale indicates lowest to highest value of BLUP of bunch number. (PPTX 289 kb)

## Abbreviations

ABW: Average bunch weight
BN: Bunch number
BLUP: Best linear unbiased predictor
FFB: Fresh fruit bunch yield
GA: Heterotic group A
GB: Heterotic group B
GCA: General combining ability
IBD: Identity-by-descent
LRT: Log-likelihood ratio test
QTL: Quantitative trait locus
RRS: Reciprocal recurrent selection
SCA: Specific combining ability
SSR: Simple sequence repeats
