# Supplementary material for: Mixed model approach for IBD-based QTL mapping in a complex oil palm pedigree
Source: BMC Genomics. 2015 Oct 15;16:798. doi: 10.1186/s12864-015-1985-3 (PMC4608140; doi:10.1186/s12864-015-1985-3)
Supplement: Additional file 1: Figure S1. — Pedigree of oil palm heterotic groups A (A) and B (B) individuals related to A×B progenies providing phenotypic data. Palm trees from group A originate from populations Deli (DELI) and Angola (AN), those of group B originate from populations La Mé (LAME), Yangambi (YBI), Nigeria (NI) and Sibiti (SI). Palm trees used as parents of A×B progenies are in white, and for group A pedigree, palm trees surrounded with red were reconstructed using MOLCOANC software. Figure S2. Oil palm consensus genetic map obtained from recombination information in group A and B pedigrees. Linkage groups are numbered according to Billotte et al. (2005, 2010). Map distance is given on the left in centimorgans (cM) and marker names on the right. Figure S3. Null distribution of log-likelihood ratio test in oil palm group A (green) and B (blue) for first (LRT_1,A) and second (LRT_2,B) genome scan for fresh fruit bunch weight (FFB), bunch number (BN) and average bunch weight (ABW) in heterotic group A (green lines) and B (blue lines). Figure S4. Log-likelihood ratio test (LRT) profile obtained in the first genome scan (see Material and Methods) using known (black lines) or reconstructed (green lines) pedigree of oil palm heterotic group A individuals. Figure S5. Example of QTL segregation in oil palm pedigree. Additive effects on bunch number for pedigree term (A,D) and QTLs on linkage group 9 at 69 cM (B,E) and on linkage group 15 at 117 cM (C,F) are projected on two subsets of heterotic group B pedigree, one centered on La Mé LM10T individual (A-C) and one grouping most of Yangambi individuals (D-F) . Red to green scale indicates lowest to highest value of BLUP of bunch number. (PPTX 289 kb) [file 12864_2015_1985_MOESM1_ESM.pptx]

## Slide 1
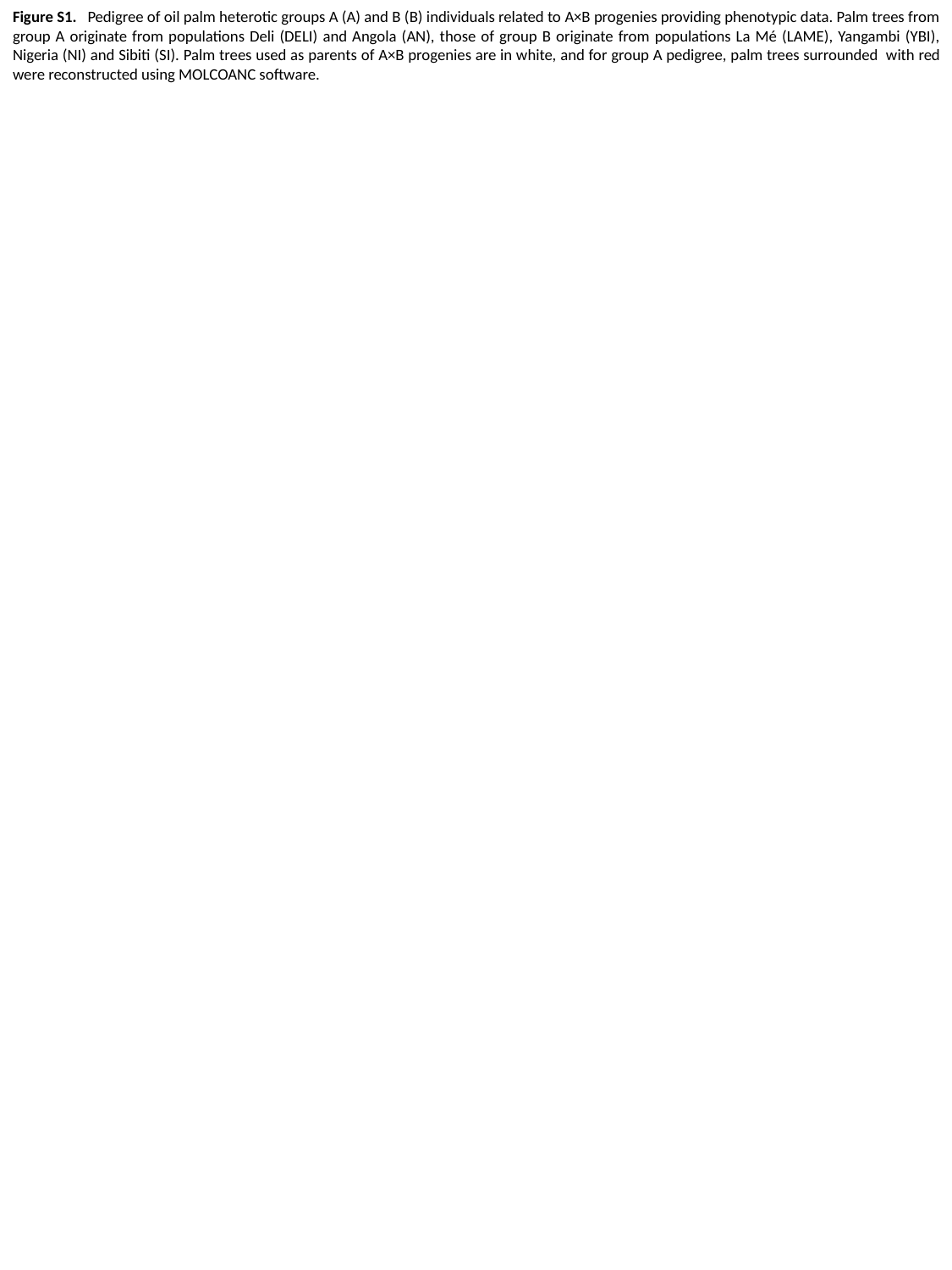

Figure S1. Pedigree of oil palm heterotic groups A (A) and B (B) individuals related to A×B progenies providing phenotypic data. Palm trees from group A originate from populations Deli (DELI) and Angola (AN), those of group B originate from populations La Mé (LAME), Yangambi (YBI), Nigeria (NI) and Sibiti (SI). Palm trees used as parents of A×B progenies are in white, and for group A pedigree, palm trees surrounded with red were reconstructed using MOLCOANC software.

## Slide 2
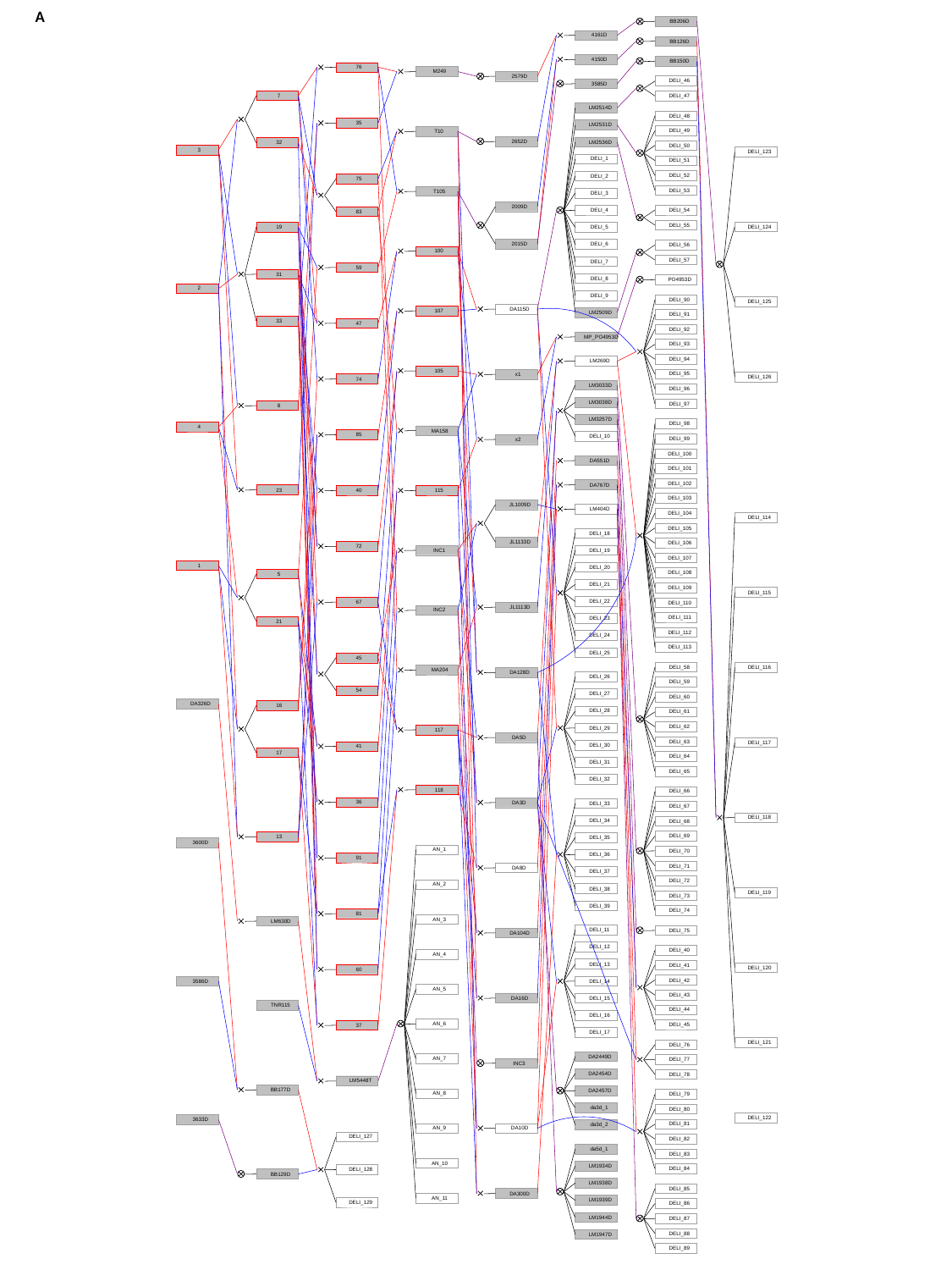

A
BB206D
4161D
BB126D
4150D
BB150D
76
M249
2579D
DELI_46
3585D
7
DELI_47
LM2514D
DELI_48
35
LM2531D
DELI_49
T10
2652D
LM2536D
32
DELI_50
3
DELI_123
DELI_1
DELI_51
DELI_52
DELI_2
75
DELI_53
T105
DELI_3
2009D
DELI_4
DELI_54
83
DELI_55
DELI_124
19
DELI_5
2015D
DELI_6
DELI_56
100
DELI_57
DELI_7
59
31
DELI_8
PO4953D
2
DELI_9
DELI_90
DELI_125
DA115D
107
LM2509D
DELI_91
33
47
DELI_92
MP_PO4953D
DELI_93
DELI_94
LM269D
105
DELI_95
x1
DELI_126
74
LM3033D
DELI_96
LM3038D
DELI_97
8
LM3257D
DELI_98
4
MA158
85
DELI_10
DELI_99
x2
DELI_100
DA551D
DELI_101
DELI_102
DA767D
23
40
115
DELI_103
JL1009D
LM404D
DELI_104
DELI_114
DELI_105
DELI_18
JL1133D
DELI_106
72
DELI_19
INC1
DELI_107
1
DELI_20
DELI_108
5
DELI_21
DELI_109
DELI_115
DELI_22
67
DELI_110
JL1113D
INC2
DELI_111
DELI_23
21
DELI_112
DELI_24
DELI_113
DELI_25
45
DELI_58
DELI_116
MA204
DA128D
DELI_26
DELI_59
54
DELI_27
DELI_60
DA326D
16
DELI_28
DELI_61
DELI_62
DELI_29
117
DA5D
DELI_63
DELI_117
DELI_30
41
17
DELI_64
DELI_31
DELI_65
DELI_32
118
DELI_66
36
DA3D
DELI_33
DELI_67
DELI_118
DELI_34
DELI_68
DELI_69
13
DELI_35
3600D
AN_1
DELI_70
DELI_36
91
DELI_71
DA8D
DELI_37
DELI_72
AN_2
DELI_38
DELI_119
DELI_73
DELI_39
DELI_74
81
AN_3
LM630D
DELI_11
DELI_75
DA104D
DELI_12
DELI_40
AN_4
DELI_13
DELI_41
DELI_120
60
DELI_42
3586D
DELI_14
AN_5
DELI_43
DA16D
DELI_15
TNR115
DELI_44
DELI_16
AN_6
DELI_45
37
DELI_17
DELI_121
DELI_76
DA2449D
AN_7
DELI_77
INC3
DA2454D
DELI_78
LM5448T
BB177D
DA2457D
AN_8
DELI_79
da3d_1
DELI_80
DELI_122
3633D
DELI_81
da3d_2
AN_9
DA10D
DELI_127
DELI_82
da5d_1
DELI_83
AN_10
LM1934D
DELI_84
DELI_128
BB129D
LM1938D
DELI_85
DA300D
AN_11
LM1939D
DELI_129
DELI_86
LM1944D
DELI_87
DELI_88
LM1947D
DELI_89

## Slide 3
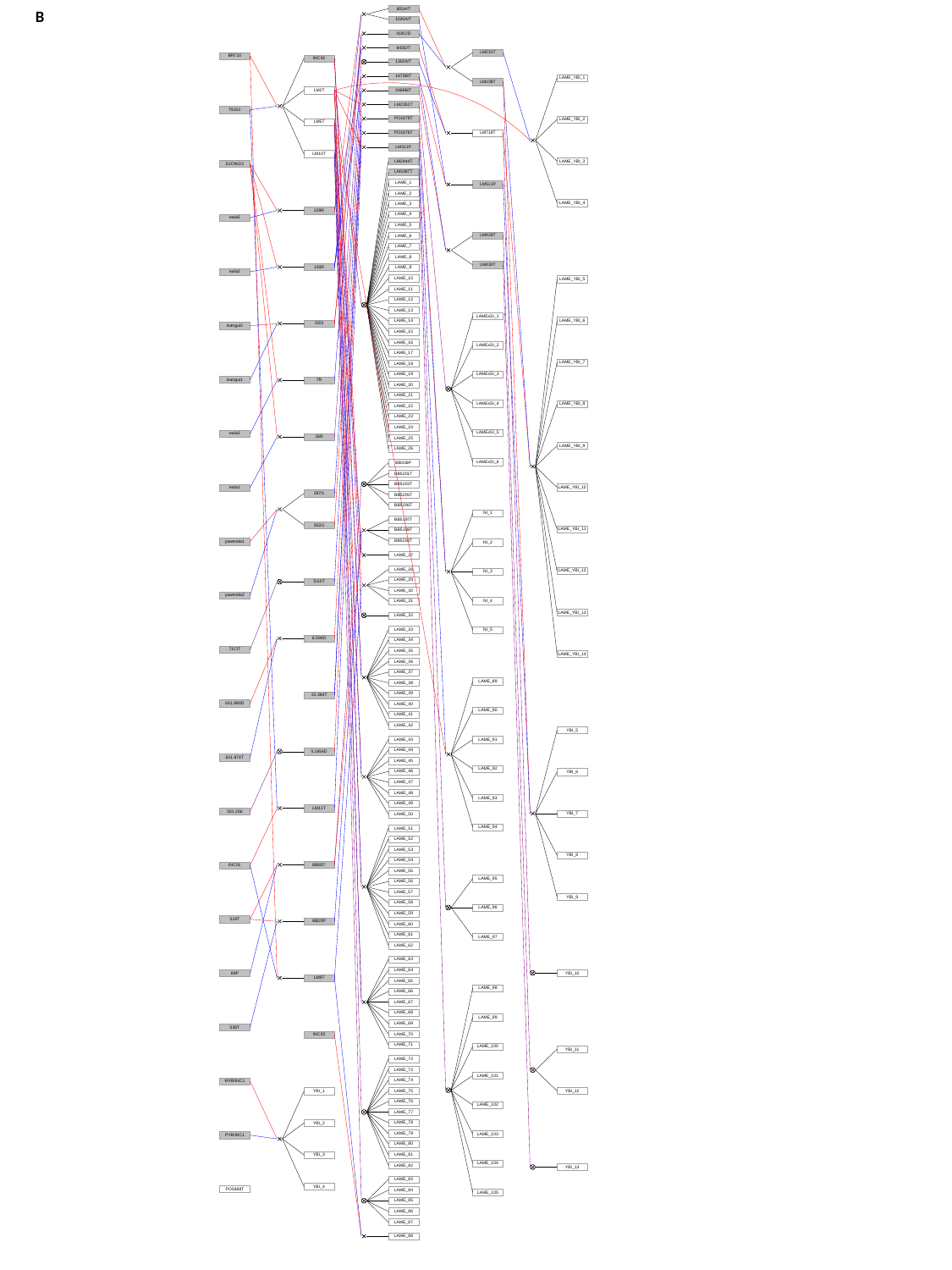

B

## Slide 4
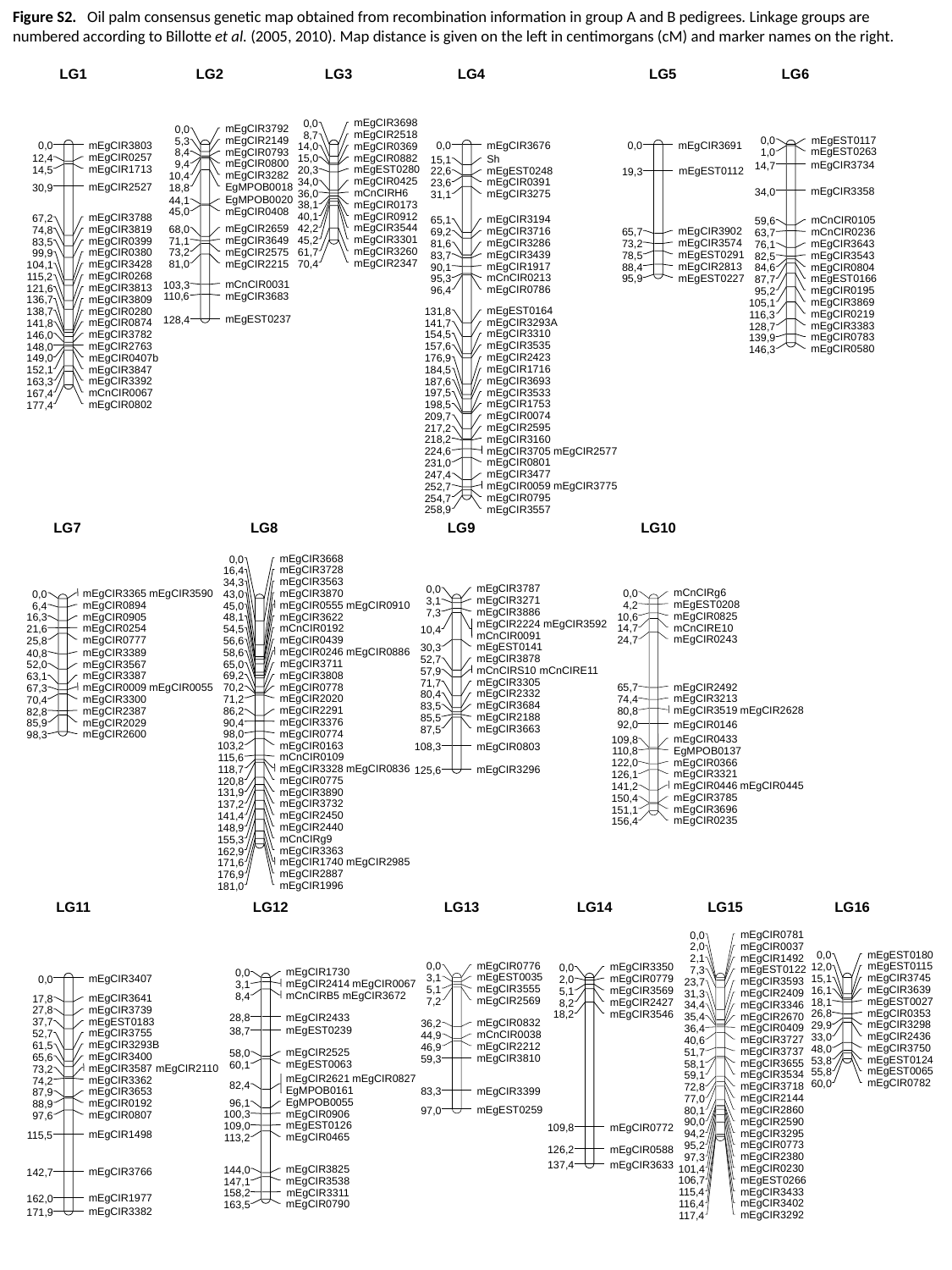

Figure S2. Oil palm consensus genetic map obtained from recombination information in group A and B pedigrees. Linkage groups are numbered according to Billotte et al. (2005, 2010). Map distance is given on the left in centimorgans (cM) and marker names on the right.
LG1
mEgCIR3803
0,0
mEgCIR0257
12,4
mEgCIR1713
14,5
mEgCIR2527
30,9
mEgCIR3788
67,2
mEgCIR3819
74,8
mEgCIR0399
83,5
mEgCIR0380
99,9
mEgCIR3428
104,1
mEgCIR0268
115,2
mEgCIR3813
121,6
mEgCIR3809
136,7
mEgCIR0280
138,7
mEgCIR0874
141,8
mEgCIR3782
146,0
mEgCIR2763
148,0
mEgCIR0407b
149,0
mEgCIR3847
152,1
mEgCIR3392
163,3
mCnCIR0067
167,4
mEgCIR0802
177,4
LG2
mEgCIR3792
0,0
mEgCIR2149
5,3
mEgCIR0793
8,4
mEgCIR0800
9,4
mEgCIR3282
10,4
EgMPOB0018
18,8
EgMPOB0020
44,1
mEgCIR0408
45,0
mEgCIR2659
68,0
mEgCIR3649
71,1
mEgCIR2575
73,2
mEgCIR2215
81,0
mCnCIR0031
103,3
mEgCIR3683
110,6
mEgEST0237
128,4
LG3
mEgCIR3698
0,0
mEgCIR2518
8,7
mEgCIR0369
14,0
mEgCIR0882
15,0
mEgEST0280
20,3
mEgCIR0425
34,0
mCnCIRH6
36,0
mEgCIR0173
38,1
mEgCIR0912
40,1
mEgCIR3544
42,2
mEgCIR3301
45,2
mEgCIR3260
61,7
mEgCIR2347
70,4
LG4
mEgCIR3676
0,0
Sh
15,1
mEgEST0248
22,6
mEgCIR0391
23,6
mEgCIR3275
31,1
mEgCIR3194
65,1
mEgCIR3716
69,2
mEgCIR3286
81,6
mEgCIR3439
83,7
mEgCIR1917
90,1
mCnCIR0213
95,3
mEgCIR0786
96,4
mEgEST0164
131,8
mEgCIR3293A
141,7
mEgCIR3310
154,5
mEgCIR3535
157,6
mEgCIR2423
176,9
mEgCIR1716
184,5
mEgCIR3693
187,6
mEgCIR3533
197,5
mEgCIR1753
198,5
mEgCIR0074
209,7
mEgCIR2595
217,2
mEgCIR3160
218,2
mEgCIR3705
mEgCIR2577
224,6
mEgCIR0801
231,0
mEgCIR3477
247,4
mEgCIR0059
mEgCIR3775
252,7
mEgCIR0795
254,7
mEgCIR3557
258,9
LG5
mEgCIR3691
0,0
mEgEST0112
19,3
mEgCIR3902
65,7
mEgCIR3574
73,2
mEgEST0291
78,5
mEgCIR2813
88,4
mEgEST0227
95,9
LG6
mEgEST0117
0,0
mEgEST0263
1,0
mEgCIR3734
14,7
mEgCIR3358
34,0
mCnCIR0105
59,6
mCnCIR0236
63,7
mEgCIR3643
76,1
mEgCIR3543
82,5
mEgCIR0804
84,6
mEgEST0166
87,7
mEgCIR0195
95,2
mEgCIR3869
105,1
mEgCIR0219
116,3
mEgCIR3383
128,7
mEgCIR0783
139,9
mEgCIR0580
146,3
LG7
mEgCIR3365
mEgCIR3590
0,0
mEgCIR0894
6,4
mEgCIR0905
16,3
mEgCIR0254
21,6
mEgCIR0777
25,8
mEgCIR3389
40,8
mEgCIR3567
52,0
mEgCIR3387
63,1
mEgCIR0009
mEgCIR0055
67,3
mEgCIR3300
70,4
mEgCIR2387
82,8
mEgCIR2029
85,9
mEgCIR2600
98,3
LG8
mEgCIR3668
0,0
mEgCIR3728
16,4
mEgCIR3563
34,3
mEgCIR3870
43,0
mEgCIR0555
mEgCIR0910
45,0
mEgCIR3622
48,1
mCnCIR0192
54,5
mEgCIR0439
56,6
mEgCIR0246
mEgCIR0886
58,6
mEgCIR3711
65,0
mEgCIR3808
69,2
mEgCIR0778
70,2
mEgCIR2020
71,2
mEgCIR2291
86,2
mEgCIR3376
90,4
mEgCIR0774
98,0
mEgCIR0163
103,2
mCnCIR0109
115,6
mEgCIR3328
mEgCIR0836
118,7
mEgCIR0775
120,8
mEgCIR3890
131,9
mEgCIR3732
137,2
mEgCIR2450
141,4
mEgCIR2440
148,9
mCnCIRg9
155,3
mEgCIR3363
162,9
mEgCIR1740
mEgCIR2985
171,6
mEgCIR2887
176,9
mEgCIR1996
181,0
LG9
mEgCIR3787
0,0
mEgCIR3271
3,1
mEgCIR3886
7,3
mEgCIR2224
mEgCIR3592
10,4
mCnCIR0091
mEgEST0141
30,3
mEgCIR3878
52,7
mCnCIRS10
mCnCIRE11
57,9
mEgCIR3305
71,7
mEgCIR2332
80,4
mEgCIR3684
83,5
mEgCIR2188
85,5
mEgCIR3663
87,5
mEgCIR0803
108,3
mEgCIR3296
125,6
LG10
mCnCIRg6
0,0
mEgEST0208
4,2
mEgCIR0825
10,6
mCnCIRE10
14,7
mEgCIR0243
24,7
mEgCIR2492
65,7
mEgCIR3213
74,4
mEgCIR3519
mEgCIR2628
80,8
mEgCIR0146
92,0
mEgCIR0433
109,8
EgMPOB0137
110,8
mEgCIR0366
122,0
mEgCIR3321
126,1
mEgCIR0446
mEgCIR0445
141,2
mEgCIR3785
150,4
mEgCIR3696
151,1
mEgCIR0235
156,4
LG11
mEgCIR3407
0,0
mEgCIR3641
17,8
mEgCIR3739
27,8
mEgEST0183
37,7
mEgCIR3755
52,7
mEgCIR3293B
61,5
mEgCIR3400
65,6
mEgCIR3587
mEgCIR2110
73,2
mEgCIR3362
74,2
mEgCIR3653
87,9
mEgCIR0192
88,9
mEgCIR0807
97,6
mEgCIR1498
115,5
mEgCIR3766
142,7
mEgCIR1977
162,0
mEgCIR3382
171,9
LG12
mEgCIR1730
0,0
mEgCIR2414
mEgCIR0067
3,1
mCnCIRB5
mEgCIR3672
8,4
mEgCIR2433
28,8
mEgEST0239
38,7
mEgCIR2525
58,0
mEgEST0063
60,1
mEgCIR2621
mEgCIR0827
82,4
EgMPOB0161
EgMPOB0055
96,1
mEgCIR0906
100,3
mEgEST0126
109,0
mEgCIR0465
113,2
mEgCIR3825
144,0
mEgCIR3538
147,1
mEgCIR3311
158,2
mEgCIR0790
163,5
LG13
mEgCIR0776
0,0
mEgEST0035
3,1
mEgCIR3555
5,1
mEgCIR2569
7,2
mEgCIR0832
36,2
mCnCIR0038
44,9
mEgCIR2212
46,9
mEgCIR3810
59,3
mEgCIR3399
83,3
mEgEST0259
97,0
LG14
mEgCIR3350
0,0
mEgCIR0779
2,0
mEgCIR3569
5,1
mEgCIR2427
8,2
mEgCIR3546
18,2
mEgCIR0772
109,8
mEgCIR0588
126,2
mEgCIR3633
137,4
LG15
mEgCIR0781
0,0
mEgCIR0037
2,0
mEgCIR1492
2,1
mEgEST0122
7,3
mEgCIR3593
23,7
mEgCIR2409
31,3
mEgCIR3346
34,4
mEgCIR2670
35,4
mEgCIR0409
36,4
mEgCIR3727
40,6
mEgCIR3737
51,7
mEgCIR3655
58,1
mEgCIR3534
59,1
mEgCIR3718
72,8
mEgCIR2144
77,0
mEgCIR2860
80,1
mEgCIR2590
90,0
mEgCIR3295
94,2
mEgCIR0773
95,2
mEgCIR2380
97,3
mEgCIR0230
101,4
mEgEST0266
106,7
mEgCIR3433
115,4
mEgCIR3402
116,4
mEgCIR3292
117,4
LG16
mEgEST0180
0,0
mEgEST0115
12,0
mEgCIR3745
15,1
mEgCIR3639
16,1
mEgEST0027
18,1
mEgCIR0353
26,8
mEgCIR3298
29,9
mEgCIR2436
33,0
mEgCIR3750
48,0
mEgEST0124
53,8
mEgEST0065
55,8
mEgCIR0782
60,0

## Slide 5
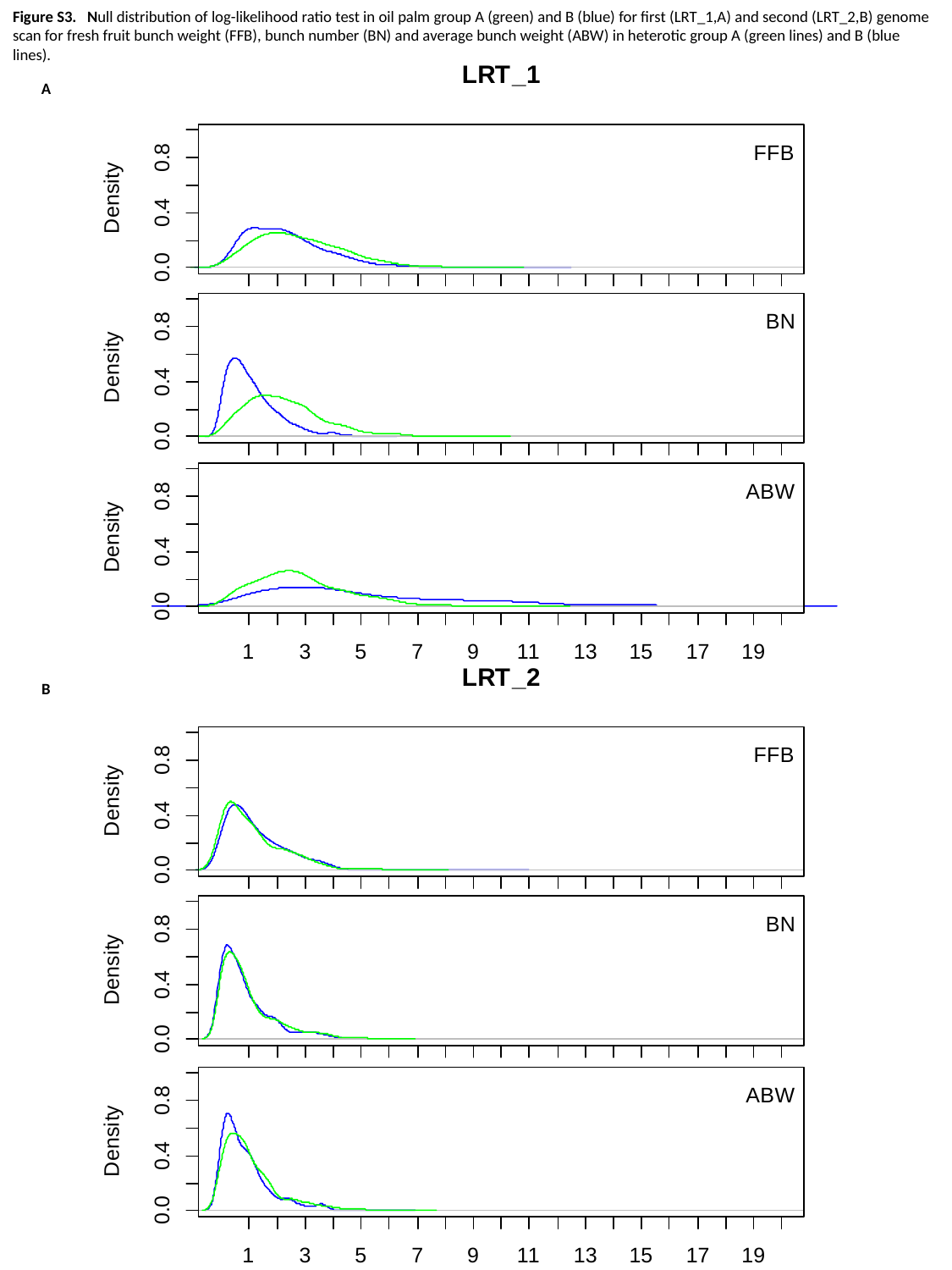

Figure S3. Null distribution of log-likelihood ratio test in oil palm group A (green) and B (blue) for first (LRT_1,A) and second (LRT_2,B) genome scan for fresh fruit bunch weight (FFB), bunch number (BN) and average bunch weight (ABW) in heterotic group A (green lines) and B (blue lines).
A
B

## Slide 6
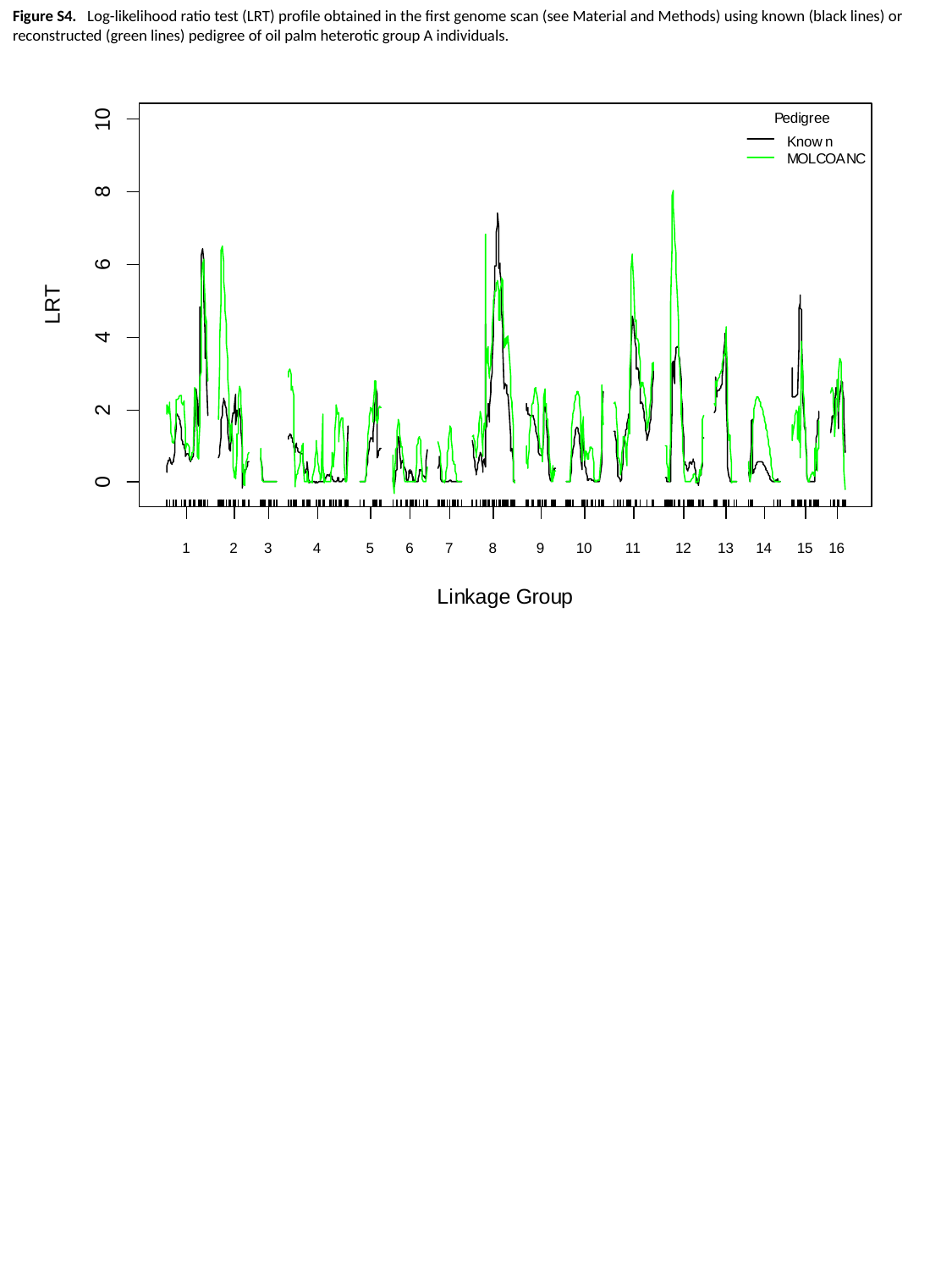

Figure S4. Log-likelihood ratio test (LRT) profile obtained in the first genome scan (see Material and Methods) using known (black lines) or reconstructed (green lines) pedigree of oil palm heterotic group A individuals.

## Slide 7
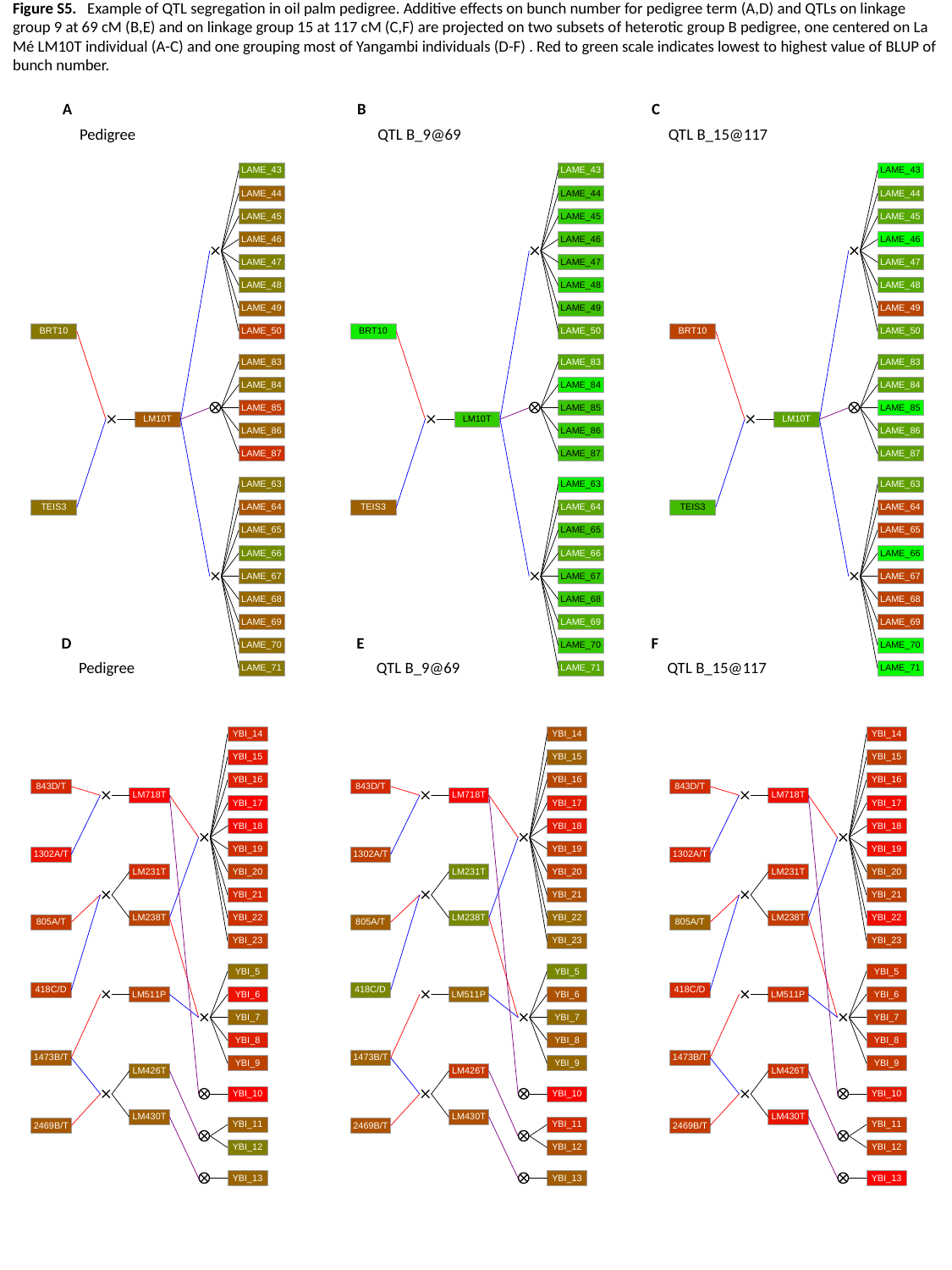

Figure S5. Example of QTL segregation in oil palm pedigree. Additive effects on bunch number for pedigree term (A,D) and QTLs on linkage group 9 at 69 cM (B,E) and on linkage group 15 at 117 cM (C,F) are projected on two subsets of heterotic group B pedigree, one centered on La Mé LM10T individual (A-C) and one grouping most of Yangambi individuals (D-F) . Red to green scale indicates lowest to highest value of BLUP of bunch number.
A
B
C
Pedigree
 QTL B_9@69
QTL B_15@117
D
E
F
Pedigree
 QTL B_9@69
QTL B_15@117
